# Tools to Dissect Lipid Droplet Regulation, Players, and Mechanisms

**DOI:** 10.1021/acschembio.4c00835

**Published:** 2025-03-04

**Authors:** Jinmin Liu, Yimon Aye

**Affiliations:** † University of Oxford, Oxford OX1 3TA, United Kingdom

## Abstract

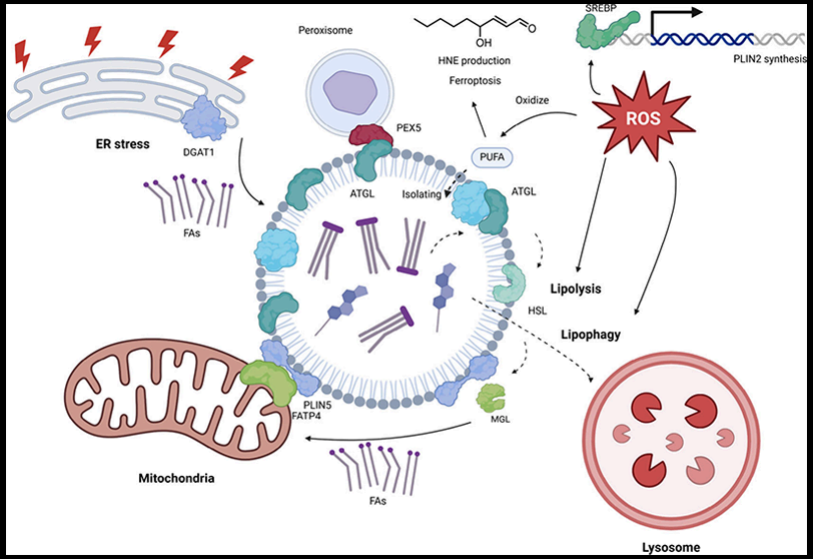

Spurred by the authors’ own recent discovery of reactive metabolite-regulated nexuses involving lipid droplets (LDs), this perspective discusses the latest knowledge and multifaceted approaches toward deconstructing the function of these dynamic organelles, LD-associated localized signaling networks, and protein players. Despite accumulating knowledge surrounding protein families and pathways of conserved importance for LD homeostasis surveillance and maintenance across taxa, much remains to be understood at the molecular level. In particular, metabolic stress-triggered contextual changes in LD-proteins’ localized functions, crosstalk with other organelles, and feedback signaling loops and how these are specifically rewired in disease states remain to be illuminated with spatiotemporal precision. We hope this perspective promotes an increased interest in these essential organelles and innovations of new tools and strategies to better understand context-specific LD regulation critical for organismal health.

## 1 Prelude

Once considered an inert structure in cells, lipid droplets (LDs) are now widely recognized as dynamic organelles essential for numerous aspects of organism physiology. LDs comprise triacylglycerol, sterol esters, and various LD-associated proteins. They feature a unique structure, with a hydrophobic core surrounded by a phospholipid monolayer.^1^ Beyond managing lipid homeostasis and nutrient stores, LDs buffer toxic lipid peroxidation products and further play functional roles through contact with other cellular organelles. As recent comprehensive reviews have covered the diverse biological and biochemical facets of LDs in physiology and disease, our perspective only briefly touches on these aspects. Conversely, a stronger emphasis is placed on classical methods as well as emerging tools used to peer into LD-specific functions and signaling networks. Some representative examples are discussed, highlighting strengths and limitations within each method of approach. Where applicable, we provide perspectives on outstanding questions and how interdisciplinary strategies could aid these investigations.

## 2 LD Metabolism

Understanding the molecular mechanisms involved in LD metabolism is crucial for comprehending how cells regulate lipid homeostasis, particularly in metabolic diseases including obesity, diabetes, and fatty liver disease.^2^ As with all organelles, regulated mechanisms are in place for LD build-up and breakdown. Below, we overview key aspects of LD metabolism while referring to existing in-depth reviews for further reading.^1,3−6^

### 2.1 Anabolism

**Figure 1:**
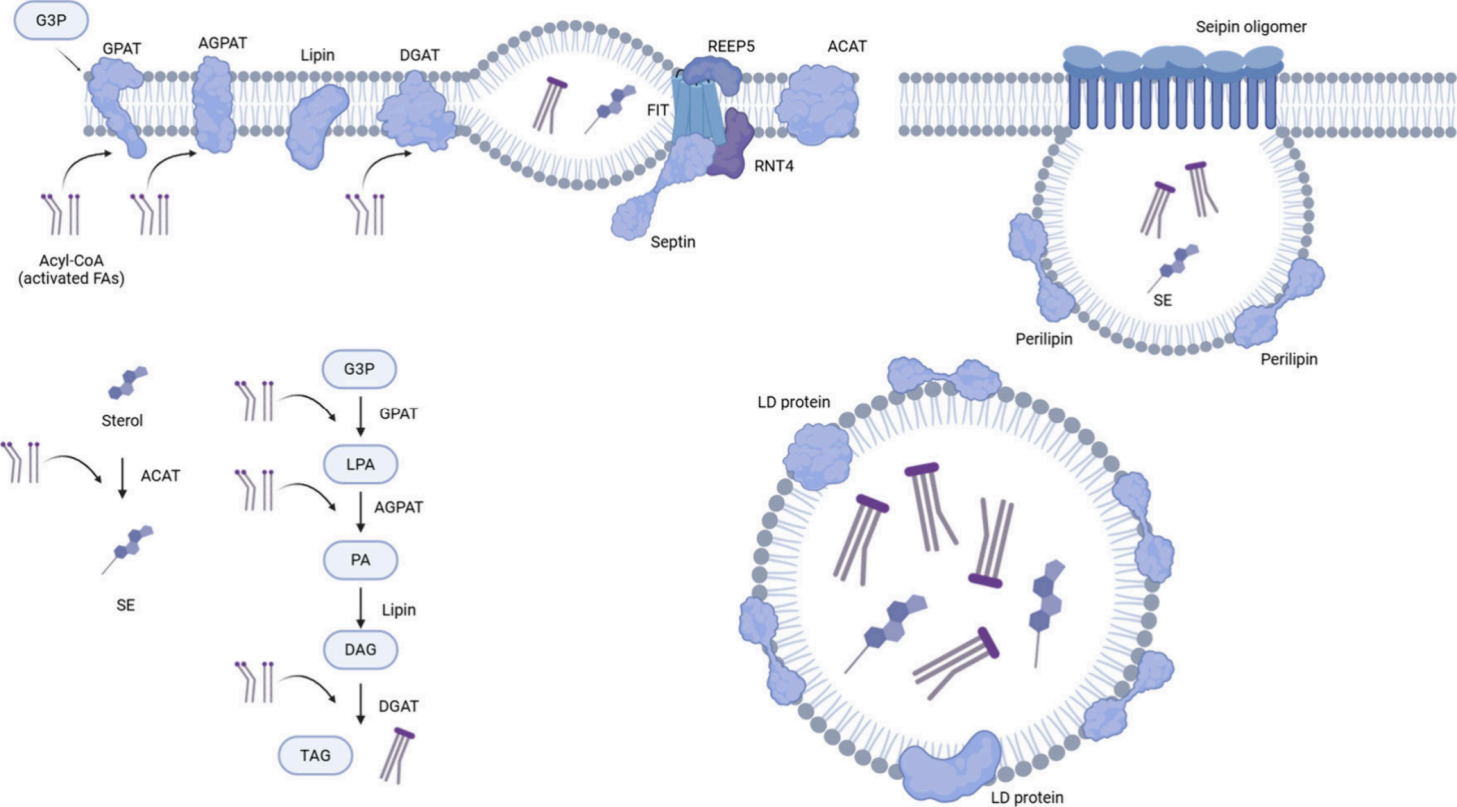
Biogenesis of lipid droplets (LDs). LD biogenesis involves the synthesis of triacylglycerols (TAG) and sterol esters (SE). The TAG synthesis starts with glycerol-3-phosphate (G3P) through several enzymatic reactions, with activated fatty acids (acyl-CoA) as substrate. Glycerol-3-phosphate acyltransferase (GPAT) is the rate-limiting enzyme that transfers G3P to lysophosphatidic acid (LPA), followed by 1-acylglycerol-3-phosphate-*O*-acyltransferase (AGPAT) that catalyzes generation of phosphatidic acid (PA), lipin to produce diacylglycerols (DAG), and diacylglycerol acyltransferases (DGATs), feeding toward TAG synthesis. On the other hand, cholesterol and Acyl-CoA form sterol esters (SEs) catalyzed by Acyl-CoA cholesterol *O*-acyltransferases (ACATs). Newly synthesized TAG accumulates in the monolayer of the endoplasmic reticulum (ER), forming nascent lipid droplets by the assistance of periliplin (PLIN), fat-inducing transcript (FIT) proteins, seipin oligomer, and their cofactors.

LD biogenesis is initiated through multistep enzymatic syntheses of neutral lipids, primarily triacylglycerols (TAG) and sterol esters (SE) (Figure 1). These enzymes for both TAG and SE biosyntheses are conserved across eukaryotes. Interestingly, beyond higher-order organisms including mice, fish, worms and fruit flies, diacylglycerol acyltransferases (DGATs) for TAG synthesis are conserved even in plants^7^ and algae.^8^ Key SE-synthesis enzymes, Acyl-CoA cholesterol *O*-acyltransferases (ACATs), are conserved from yeast to humans. These TAG and SE biosynthesis occur in endoplasmic reticulum (ER). Once these newly synthesized neutral lipids reach a certain concentration in the ER membrane, the encapsulated TAG and SE form an “oil lens” between two phospholipid monolayers. Although growing reports hint that LD budding does not exclusively rely on TAG or SE synthesis, a deficiency in either pathway interferes with the LD number and size. The current model suggests that the so-called “lens” formation and the nucleation of these neutral lipids are spontaneous, driven by the need to minimize interactions with the charged phospholipids or proteins of the ER membrane.^9^ Nascent LD assembly is additionally influenced by the local proteome and biophysical factors, including lipid rigidity, lipid composition,^10^ membrane tension,^11^ and ER structure (sheets versus tubules^12^), among others.^13^ Although the precise mechanism of LD budding is not yet fully understood, several vital proteins have been identified, especially seipin,^14^ perilipins, and fat storage-inducing transmembrane (FITM) proteins,^15^ that collectively enable the LD budding sites. Recent studies show FITM interacting with ER tubule-forming proteins (REEP5, RTN4) and cytoskeletal septins. These proteins form puncta along the tubules, colocalizing with nascent LDs.^16^

### 2.2 Catabolism

**Figure 2:**
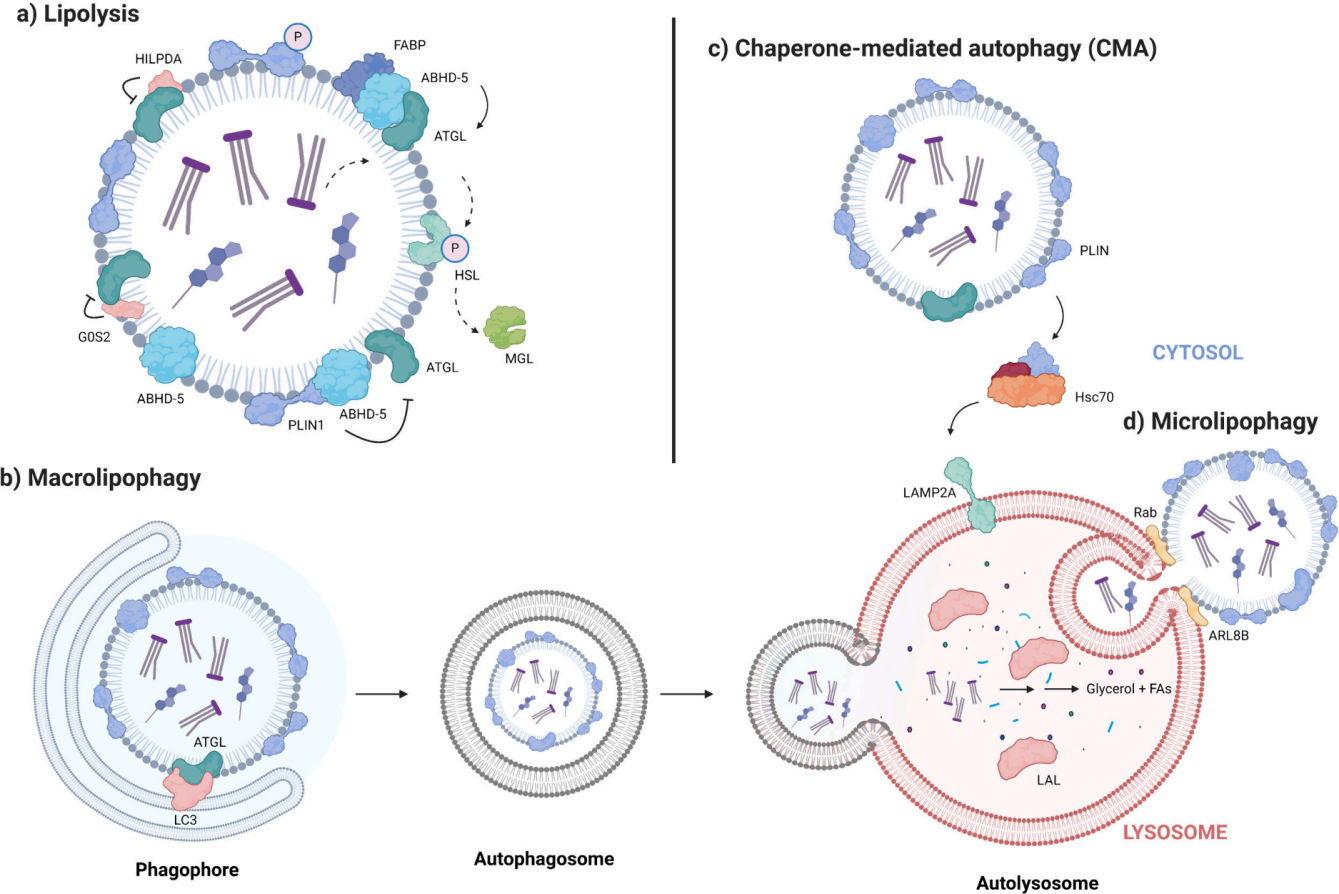
Degradation of LDs. (a) Neutral lipolysis involves three primary lipases (ATGL, HSL, MGL) that digest TAG. Multiple proteins regulate the activity of adipose triglyceride lipase (ATGL). The full activity of ATGL requires binding of ABHD-5, and the interaction between periliplin and ABHD-5 inhibits the association between ATGL and ABHD-5. FABP binding to ABHD-5 promotes ATGL activity, but the G0/G1 switch gene-2 (G0S2) and hypoxia-induced LD-associated protein (HILPDA) inhibit ATGL. On the other hand, activation and translocation to LDs of hormone-sensitive lipase (HSL) are regulated by phosphorylation. Unlike ATGL or HSL, which are closely associated with lipid droplets, MGL functions primarily within cell membranes and the cytoplasm. (b) In macrolipophagy, LDs are engulfed by a double-membrane structure known as the autophagosome. ATGL and LC3 interaction promotes the growth of autophagosomes encapsulating LDs. After the fusion to the lysosome, the TAG and sterol esters (SE) are digested by lysosomal acid lipase (LAL). (c, d) Without the formation of the autophagosome, LD components can be delivered to the lysosome and digested by Hsc70, LAMP2A mediated autophagy, or Rab, ADP ribosylation factors like GTPase 8B (ARL8B) mediated direct contact (microlipophagy).

LD catabolism necessitates breaking down both lipids and proteins. Lipid degradation occurs via two main pathways: lipolysis (specifically, neutral enzymatic hydrolysis) and lipophagy^4^ (autophagic components associated mechanism) (Figure 2). Conversely, LD-associated protein turnover occurs via ubiquitin-proteasome system (UPS) and autophagy (including chaperone-mediated autophagy and macroautophagy).^17^ Given the strong link between lipid levels and the functional importance of LDs, we discuss the latest knowledge underpinning lipolysis and lipophagy. We refer to existing in-depth reviews for regulated degradation of LD-associated proteins.

#### 2.2.1 Lipolysis

Lipolysis (specifically, neutral lipolysis) involves a series of enzymatic reactions converting TAG into free fatty acids and glycerol, for ATP production by β-oxidation occurring in the peroxisome or/and mitochondria^18^ (Figure 2a). SE degradation plays a more critical role in maintaining sterol homeostasis as opposed to regulating LD size and number. Although much is now known about TAG degradation, transport mechanisms guiding key enzymes regulating lipolysis, such as adipose triglyceride lipase (ATGL, PNPLA2), from ER to lipid droplets remain poorly understood. Indeed, locale-specific *functions* of canonical localized proteins remain a major unsolved problem in the broader field, beyond LD biology, largely due to the lack of tools to map such nuanced changes in locale-specific activities (see discussions later in the perspective). As with LD-anabolism, TAG and SE hydrolyses are highly conserved biochemical pathways across plants,^19^ yeast,^20^*C. elegans*,^21^ and humans. For example, in canonical TAG degradation, TAG is sequentially hydrolyzed by ATGL, hormone-sensitive lipase (HSL), and monoacylglycerol lipase (MGL), and their orthologs are conserved across different organisms. ABHD5/CGI-58/LID-1, first identified in *C. elegans*, is a conserved key regulator that activates ATGL, enabling it to reach full enzymatic activity and initiate TAG hydrolysis.

#### 2.2.2 Lipophagy

The discovery of (macro)lipophagy—a form of macroautophagy, that transports individual LDs to lysosomes—in 2009, brought a new dimension into LD breakdown involving autophagic components (Figure 2b). Since then, inventory of novel players supporting lipophagy, particularly LD receptor proteins required for functional interaction with autophagosomal membrane residents, continues to grow. Representative autophagic components, such as LC3 (atg8 in yeast, lgg in *C. elegans*), Beclin-1 (atg6 in yeast, bec-1 in *C. elegans*), interact with LD-resident proteins, including ATGL and other PNPLA family members. Ubiquitinated LD-protein receptors, such as SQSTM1/p62 (sqst-1 in *C. elegans*), as well as small GTPase Rab family, are also highly conserved and play crucial roles in macrolipophagy.

Two additional lipophagy mechanisms have also emerged recently (Figure 2c,d)—microlipophagy and chaperone-mediated autophagy (CMA), referred to as “direct lysosomal LD degradation” as they degrade partial components of LDs, in lieu of whole LDs as in macrolipophagy (Figure 2b).^22^ Although CMA pathway is primarily known for degrading key LD-associated proteins, including PLIN2, PLIN3^23^ and PLIN5^24^ (essential for LD-organization, scaffolding, and metabolic functions), CMA also plays a critical role in maintaining LD homeostasis. CMA pathway inhibition or deficiency in mice liver causes abnormal lipid accumulation and lipid homeostasis imbalance.^25^ Recently, microlipophagy in mammalian cells was demonstrated using hepatocytes, showing its independence from autophagosomal intermediates.^26^ In microlipophagy, once LD-associated lipids are transferred to the lysosomes, they are degraded by lysosomal acid lipase (LAL, LIPA), the only known enzymes capable of breaking down TAG, DAG, cholesteryl esters (CE), and retinyl esters under acidic conditions.^27^

These emerging lipophagy pathways provide novel insights into complex lipid degradation mechanisms. Nonetheless, detailed regulatory processes and associated protein components, especially in the context of LD dysregulation, still require further investigation.

## 3 Additional Regulators of LD Metabolism

**Figure 3:**
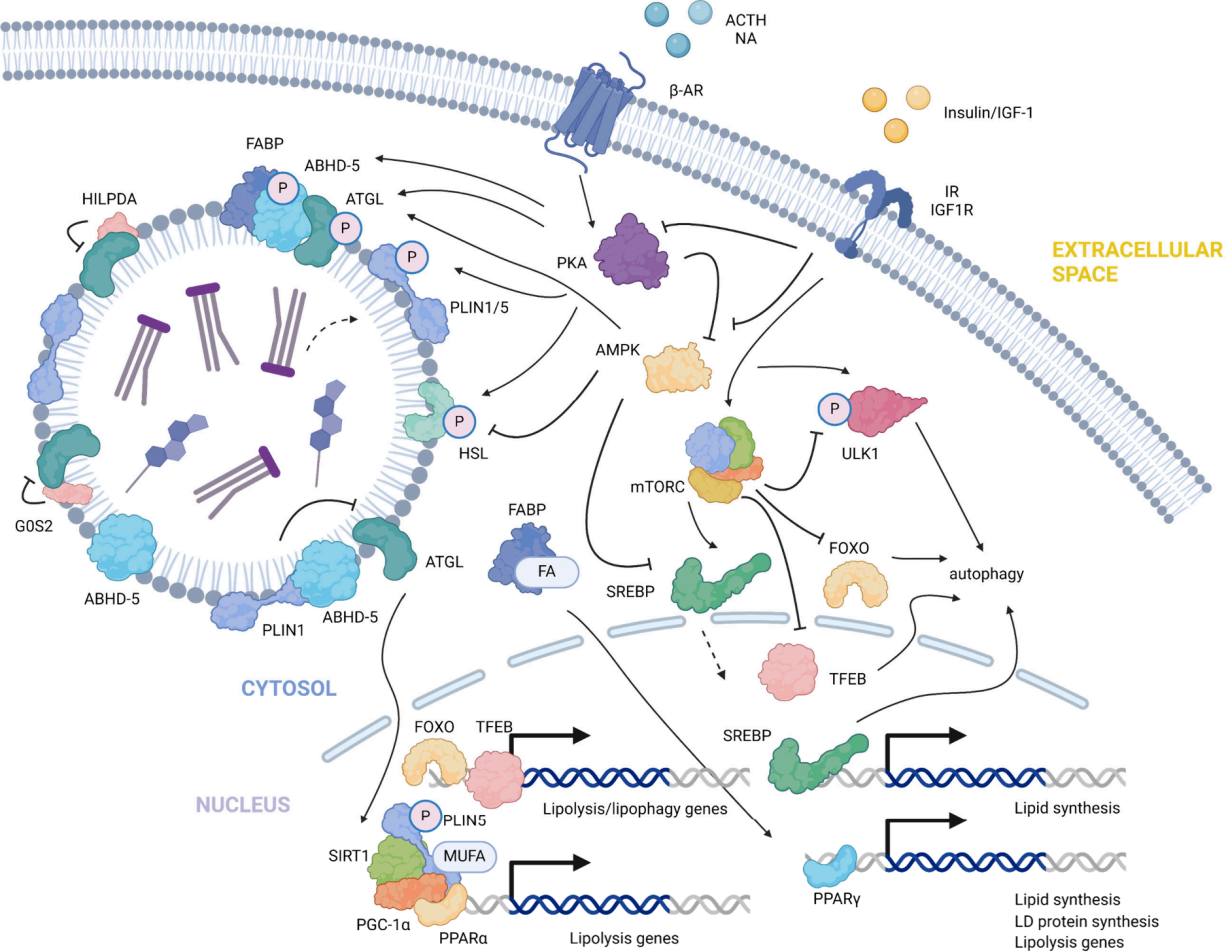
Regulatory pathways of LDs. Sophisticated networks regulate the expression and localized activities of LD proteins. From the perspective of post-translational modifications, phosphorylation of ABHD-5, ATGL, and PLINs by protein kinase A (PKA) is considered obligatory for achieving ATGL activity and increased lipolysis. Phosphorylation of PLINs induces disassociation of ABHD-5 and PLIN and activates the interaction between ABHD-5 and ATGL. The role of AMPK is diverse and remains controversial: its high activity increases autophagy and suppresses lipid biosynthesis. However, AMPK also reportedly inhibits HSL activity. Transcription factors such as SIRT1, PGC-1α, FOXO, SREBP, and PPAR control the expression of genes related to lipid metabolism. Upstream regulators, e.g., mTOR and AMPK, influence these factors. The free fatty acids (FA) can change the activity of these factors by binding to PLIN5 or FABP, which interacts with SIRT1 and PPARs. Hormones additionally constitute critical upstream factors regulating lipid metabolism. The binding of noradrenalin (NA) or adrenocorticotropic hormone (ACTH) to β-adrenergic G-protein-coupled receptors activates PKA significantly. Activation of insulin/IGF-1 pathway increases LD biosynthesis and downregulates lipid degradation by mTOR and AMPK.

Beyond the intrinsic enzymatic processes governing LD generation and degradation described above, LDs are subjected to regulation by diverse hormones, stress responses, and signaling pathways, and are under transcriptional and posttranslational control. (Figure 3)

### 3.1 Nonenzymatic Regulatory Factors

Catecholamines are classical lipid-degradation activators, and so are glucocorticoids, thyroid hormones, eicosanoids, atrial natriuretic peptides, growth hormones, and interleukins.^28^ Insulin, in contrast, is the primary inhibitor of lipid degradation. These hormones initiate different kinase-mediated pathways, such as mTORC, AKT, or AMPK (discussed further below), which then regulate multiple downstream targets including the sterol regulatory element-binding protein 1 (SREBP1) and/or forkhead box protein O (FOXO) for regulation of lipid biosynthesis enzymes and LD-associated proteins, as well as the activities for lipid-degradation enzymes.

### 3.2 Enzymatic Post-translational Modifications (E-PTMs)

Multiple E-PTMs on LD-associated proteins and enzymes involved in lipid biosynthesis and degradation have been identified. For example, N-terminal acetylation initiates the degradation process of PLIN2,^29^ (one of the members of LD-scaffolding protein PLIN family), *S*-acylation is essential for achieving enzymatically active ATGL^30^ (the key enzyme catalyzing the lipolysis first-step), and glycosylation of seipin^31^ (an integral membrane-protein supporting ER-LD-contact sites) controls the size of LDs. All of these proteins are essential for maintaining LD homeostasis. Interestingly, lipidation-based E-PTMs also play a role in protein localization and targeting of LDs. For instance, *N*-myristoylation of ANKRD22^32^ and prenylation of aldehyde dehydrogenase ALDH3B2,^33^ are lipid-modifications that assist trafficking of such proteins to LDs.

Among E-PTMs involved in LD-metabolism, phosphorylation is the most extensively studied, involving various kinases that modulate downstream enzyme activation/inhibition, subcellular translocation, and protein–protein interactions. Phosphorylation of lipid-biosynthesis enzymes is less frequently reported compared to that of lipid-degradation enzymes. For instance, phosphorylation of glycerol-3-phosphate acyltransferase (GPAT) downregulates its activity,^34^ inhibiting G3P synthesis. AMPK phosphorylates ACC1 (acetyl-CoA carboxylase 1) and ACC2, thereby suppressing fatty acid synthesis required for LD anabolism. mTOR phosphorylates lipin, the key enzyme for DAG synthesis, and prevents its translocation to the nucleus, where lipin inhibits the key lipo- and steroidogenic gene transcription activator SREBP.^35^ Beyond the hormones mentioned above, AMPK is also activated by increased ratio of intracellular AMP to ATP levels caused by various types of stress including glucose deficiency, starvation,^36^ or ROS stress.^37^ In the context of catabolism, the relationship between AMPK and lipolysis remains controversial. For example, the AMPK mediates ATGL activation through phosphorylation at Ser406 but inhibits HSL translocation to LDs via Ser554 phosphorylation. However, emerging reports on AMPK-mediated phosphorylation of autophagy-related components have indicated the role of this kinase also in regulating lipophagy. mTOR, another key down-regulator of lipid degradation, acts antagonistically to AMPK that promotes LD catabolism. Conversely, phosphorylation by another kinase, PKA (following activation by cAMP, regulates key LD proteins, ATGL, HSL, PLINs, ATGL, and ABHD5), induces lipolysis. Contradictory reports surrounding LD-regulating nE-PTMs, such as AMPK’s regulation of lipolysis, further underscore the importance of careful data interpretation alongside well-controlled experimental design and contexts in dissecting these mechanistic nuances. Functional links between specific E-PTMs and LD-associated phenotypic changes thus remain poorly resolved.

### 3.3 Transcriptional and Post-transcriptional Control

Key transcriptional regulators of LD metabolism include sirtuin 1 (SIRT1), peroxisome proliferator-activated receptor-gamma coactivator-1-α (PGC-1α),^38^ forkhead box O (FOXO),^39^ sterol regulatory element-binding protein (SREBP), carbohydrate response element binding protein (ChREBP), peroxisome proliferator-activated receptors (PPARs), transcription Factor EB (TFEB), among others (Figure 3). These transcription factors are modulated by hormones or cofactor proteins, with PKA, AMPK, and mTOR being representative upstream regulators.^40^ Interestingly, many of these transcription factors cannot be simply classified as activators or suppressors. For example, PPARγ activates adipogenesis, lipid synthesis, and LD protein expression, but also induces lipolytic enzymes like HSL and ATGL. SREBP regulates numerous genes involved in lipid biosynthesis; yet it also promotes lipophagy and cholesterol mobilization. Additionally, there is also a transcriptional cross-talk between lipolysis and lipophagy. One example is where ATGL, the enzyme catalyzing lipolysis first-step, positively regulates SIRT1 activity in hepatocytes, further promoting lipophagy through SIRT1-mediated PPARα activation.^41^ Furthermore, free fatty acids bound fatty acid binding proteins (FABPs) enhance the transcriptional activities of PPARs.^42^ These results highlight that lipolysis and lipophagy are not independent processes but coordinately maintain LD homeostasis. Additionally, key LD biogenesis enzymes, for instance, DGAT and ATGL, are subjected to post-transcriptional regulation by mRNA-binding proteins such as HuR.^43^

## 4 LD Functions

**Figure 4:**
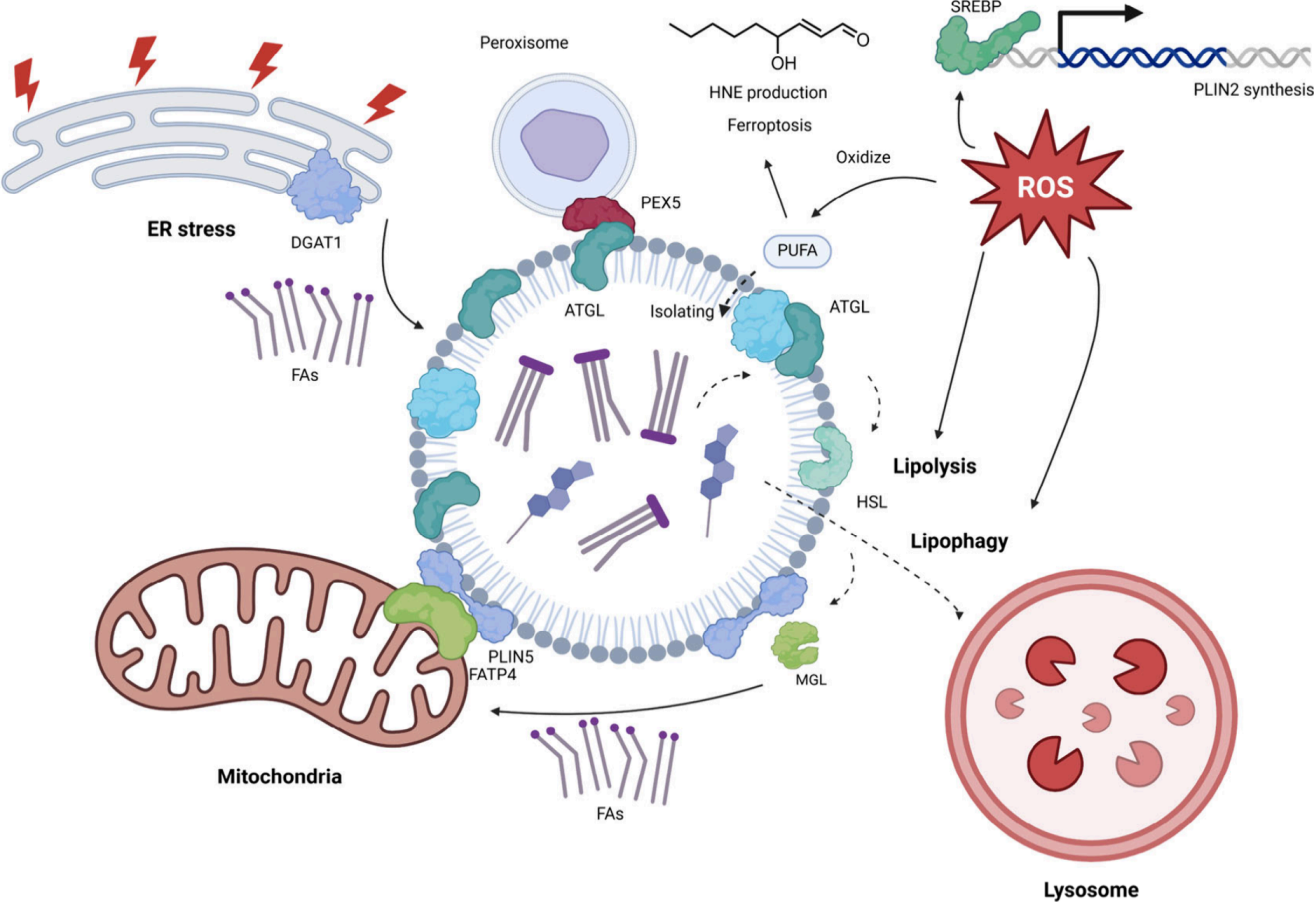
Multiple functions of LDs. DGAT1-based TAG synthesis and transfer to LD, protect the ER from lipotoxic stress (Top left). By isolating the PUFAs and excluding them from being exposed to reactive oxygen species (ROS), LDs are considered to prevent ferroptosis and the production of reactive lipid-derived electrophiles (HNE is shown here as a representative). ROS also influence the overall lipid turnover through modulation of LDs, including expression of LD proteins and lipid degradation through lipolysis and lipophagy (Top right). Through spacial contact with mitochondria, LDs efficiently deliver fatty acids to mitochondria under starvation (Bottom left). LDs also have close associations and dynamic functional connections with lysosomes and peroxisomes for lipid metabolism.

The most well-established functions of LDs are energy storage^44^ and maintaining lipid homeostasis. Yeast uses LDs to store nutrients in the nutrient-poor environment.^45^ Fatty acids stored in LDs as TAG are mobilized and released through lipolysis or lipophagy, mediated by hormones, nutrients, and cellular conditions.^1^ More recently, the importance of LDs in stress response and antilipotoxicity has gained attention^3^ (Figure 4). Substrates of TAG and SE, including free fatty acids, cholesterol, and diacyl/monoacyl-glycerol, are bioactive lipids that can be toxic in cells if their regulation is compromised.^46^ TAG and SE synthesis, the key processes in LD biogenesis, help sequester these potentially harmful lipid precursors. Thus, LD metabolism is one of most straightforward solutions to avoid lipotoxicity and widespread lipid-peroxidation-induced cellular damage, in response to various forms of stress, including ER stress, oxidative stress, and starvation.^3,5^ We focus this section on 3 key emerging contexts, namely, how LDs interplay with (i) nonenzymatic noncanonical signaling actions induced by reactive oxygen and electrophilic species (ROS/RES); (ii) ferroptotic signaling; and (iii) interorganelle signaling via membrane contact sites.

### 4.1 ROS/RES and LD Functions

Multiple triggers, including exogenous events such as exposure to reactive chemicals, hypoxia, and endogenous incidents, e.g., innate mitochondrial dysfunction and ER stress etc., elicit oxidative and electrophilic stress to cells/organisms. Once the rising RES/ROS levels overload the intrinsic detoxification systems, nondiscriminate, irreversible cellular damage is inflicted. Studies from us and others over the past few decades have enabled the field to better appreciate physiological stress-defense roles of RES^47^ and ROS,^48^ respectively. Unsurprisingly, given that LDs constitute lipid-rich environments, nuanced regulatory crosstalk between RES/ROS signaling and LD metabolism is emerging. Increased ROS exposure—induced by, for instance, exogenous addition of hydrogen peroxide; depletion of SOD1 or PDX4 (promoting endogenous abundance of superoxide)—stimulates LD biogenesis as a result of activating c-Jun N-terminal kinase (JNK) and SREBP.^49^ This process increases PLIN2 expression of relevance to PPAR signaling pathway. Under hypoxia, conditions typically considered to upregulate ROS production, hypoxia-inducible factors (HIF-1α and/or HIF-2α) drive LD biogenesis by inducing FABP^50^ and PPAR activities, lipin1 expression, and ATGL inhibition.^51^ However, non-enzyme-assisted nature of RES/ROS-based PTMs combined with their broad reactivity, makes it challenging to dissect their extraordinarily context-dependent signaling mechanisms. Additionally, ROS including hydrogen peroxide, reportedly inhibit LD degradation,^52^ while other reports have also shown ROS roles in lipolysis upregulation.^53^ Indeed, as in all contexts, precision signaling activities of ROS remain poorly understood as there is no means thus far to directly interrogate consequences of protein-specific ROS modifications in an otherwise largely unperturbed cell.

The importance of LDs for isolating and protecting free polyunsaturated fatty acids (PUFAs), the major precursor to lipid peroxidation-derived RES, such as 4-hydroxynonenal (HNE), has been widely demonstrated in multiple animals^54^ and cell models.^55,56^ HNE reportedly activates lipolysis through regulating key kinases involved in E-PTMs regulating lipolysis, such as PKA and AMPK as discussed above (Figure 4).^57^ In differentiated 3T3-L1 and primary adipocytes, bulk administration of HNE raises intracellular cyclic AMP (cAMP) levels, subsequently activates PKA, inducing phosphorylation of HSL, one of the key enzymes in lipolysis. Although HNE reportedly suppresses AMPK phosphorylation, the detailed mechanism remains unknown. Furthermore, with growing evidence of AMPK’s crucial roles in lipophagy activation, the relationships among HNE, AMPK, and LD abundance/regulation, require further investigation through carefully designed experiments and deployment of more precise tools.

On the other hand, LD biogenesis is also strongly induced by linoleate, an essential PUFA in humans, and a precursor of RES (through enzymatic as well as nonenzymatic ROS-induced RES formation), further underlining a close relationship between PUFA, diet, and LD regulation. Indeed, multifaceted mechanisms, involving both positive and negative regulation and feedback signaling of RES/ROS in LD metabolism, are increasingly recognized; yet, the contextual mechanistic details remain muddied.

### 4.2 Ferroptosis

Ferroptosis, a form of programmed cell death, characterized by the accumulation of lipid peroxides, is a pathway increasingly gaining attention from both academic and pharmaceutical research communities. Redox homeostasis and lipid peroxidation are closely linked to ferroptosis.^58^ Two key enzymes, glutathione-dependent peroxidase (GPX4), which converts toxic lipid peroxides into alcohols, and an NAD(P)H-dependent oxidoreductase, namely, ferroptosis suppressor protein 1 (FSP1), which prevents the propagation of lipid peroxidation, suppress ferroptosis. GPX4-inhibition-promoted ferroptosis is downregulated by the exogenous supplementation of cells with monounsaturated fatty acids (MUFAs),^59^ or by processes that reduce PUFA-constituted phospholipids, such as inhibition of acyl-CoA synthetase (ACSL4) that incorporates PUFA into phospholipids. Thus, ratio of PUFA and MUFA, especially their relative extent of incorporation into cell membrane phospholipids, is considered to be a critical determinant of ferroptosis.

Just as how LD biogenesis plays a key role to channel PUFAs into key components of LDs, e.g., TAG, as a means to protect the cell from PUFA-derived ROS/RES-induced cellular damage, LD anabolism likewise suppresses ferroptosis.^56^ Conversely, production of free fatty acids through lipolysis or lipophagy induces ferroptosis.^60^ Since lipid-peroxidation-derived RES, such as HNE can also be produced via both nonenzymatic (ROS-mediated) and enzymatic pathways,^61^ key specific classes of enzymes that regulate the latter path to RES production/metabolism, including cyclooxygenase (COX),^62^ lipoxygenase (LOX),^63^ and cytochrome P450 oxidoreductase (CYPs), have also been implicated in ferroptosis. Indeed, CYPs [and their coenzyme cytochrome P450 reductase (POR)] are essential drivers of ferroptosis.^64^ Unsurprisingly, RES such as HNE is increasingly appreciated as a downstream signal to propagate ferroptosis and associated pathophysiological ramifications. Nonetheless, precision context-specific roles of RES in ferroptosis remain significantly undertapped.

### 4.3 Interaction with Organelles

LDs associate with almost all organelles, including ER, Golgi, mitochondria, lysosomes, and peroxisomes.^65^ As described above, LD-lysosome interactions govern lipophagy. While ER is the site of LD biogenesis, LD themselves protect against ER stress^66^ induced by aberrant lipid metabolism,^67^ gene mutations, prion transmission, viral infections, ROS,^68^ and unfolded protein response (UPR) triggered by dysregulation of ER protein folding or ER lipid composition imbalance. LD biogenesis and associated TAG synthesis protect ER from lipotoxic stress, mitigating UPR.^69^ Through direct interaction, LDs can transfer free fatty acid to mitochondria, which fuels TCA cycle and oxidative phosphorylation. For example, LD-scaffolding protein PLIN5 and mitochondrial outer membrane protein FATP4 drive membrane contact site formation that is regulated by PKA^70^ (Figure 4). PLIN5 also interacts with Rab8a in a process regulated by AMPK activity.^71^ In human AC16 cells, phosphorylated PLIN5 induces lipolysis under starvation conditions, allowing efficient fatty acid delivery from LDs to mitochondria.^72^ Other interactor pairs, such as mitochondria protein mitofusin 2 (MFN2), LD protein PLIN1, and LD-localized Hsc70/HSPA8 can perform a similar function.^73^ Mitoguardin-2^74^ or synaptosome-associated protein 23 (SNAP23)-mediated^75^ recruitments are involved in crosstalk between LDs and mitochondria for lipid storage and phospholipid transfer. Interestingly, LD biogenesis is also intricately linked to mitochondria autophagy:^76^ accumulating fatty acids liberated from mitophagy can channel into nascent LDs as a means to decrease cellular lipotoxicity.^77^ In the context of peroxisomes, functional contacts with LDs play a role in lipolysis under fasting through PEX5-mediated ATGL translocation.^78^ LD protein M1 spastin forms a complex with peroxisomal ABCD1 to promote LD–peroxisome contact and subsequent LD-to-peroxisome FA trafficking.^79^ Coordinated interactions between peroxisomes and LDs reportedly extend lifespan in response to MUFA stress.^80^ Beyond the well-known interactions between LDs with the ER or lysosomes, other organelle interactions are less explored across different species. However, evidence exists for mitochondria-LD interactions in yeast^81^ and peroxisome-LD interactions in yeast and *C. elegans*,^78^ suggesting the conserved functional importance.

## 5 Approaches to Perturb and Probe LDs, Their Regulation, and Their Functions

With the growing realization of multidimensional roles of LDs in health and disease, the past few decades have witnessed an increasing repertoire of techniques devoted to elucidate LD composition, trafficking, signaling crosstalk, and associated regulators. Here we discuss the latest examples, while underlining strengths and limitations in each case, and provide our perspectives toward potential interdisciplinary solutions.

### 5.1 Imaging-Based Tools

#### 5.1.1 Genetically Encoded Biosensors and Epitope Tags

Accumulating interests surrounding LD biology have led to engineered LD proteins genetically encoded with fluorescent proteins (FPs),^82^ enabling researchers to examine LD-protein dynamics and colocalization. The most commonly expressed FP-labeled LD proteins include PLINs, ATGL, and DGAT2. Another popular tool is *LiveDrop*, incorporating amino acids 160–216 of the glycerolipid synthesis enzyme GPAT4, is also widely used as the FP-anchoring tag in LDs because of its small size, and hence potentially reduced invasiveness.^83^ In *C. elegans*, GFP-DHS-3 strain is widely used to monitor LD levels, as DHS-3 (HSD17B11 in humans) is one of the most abundant LD proteins, similar to PLINs. At the intact-mammal level, a knock-in mouse model generated using CRISPR/Cas9 in mouse embryonic stem cells enables nonstaining monitoring of LDs across various organs, including the liver, intestine, and brain, by expressing *TdTomato* fused to PLIN2.^84^ These genetically encoded biosensors enable real-time visualization of LD translocation, interactions with other organelles, colocalization with proteins of interest, protein recruitment/turnover, and changes in LD assembly/degradation under various conditions. Notably, LDs are heterogeneous in their protein composition, so no LD protein uniformly marks all LDs. This approach is particularly well-suited for dynamic studies of LD biology in live cells/animals. For LD proteins tagged with epitopes, immunofluorescence (IF)-imaging and immunoprecipitation (IP), commonly employed to analyze their localization and interactions. One clear downside with epitope tagging is that IF/IP-methods are limited to fixed cells/animals and cell lysates/tissue extracts. FP fusion (and in some proteins, even epitope tagging) could alter native LD-proteins’ function/activity, locale, trafficking ability, etc., and potentially LD metabolism and homeostasis maintenance. Thus, findings using these approaches would benefit from additional validations using orthogonal assays that can probe the endogenous untagged protein. Overexpression of fusion proteins could introduce potential artifacts to the native biology, which can be minimized by knocking in the tagged protein at the endogenous loci.

#### 5.1.2 Small-Molecule Dyes

Small-molecule dyes are valuable tools for visualizing both the location and quantity of LDs. The most well-known dyes for in vital staining of LD-associated lipids in cells^85^ and tissue slices, as well as in transparent model organisms^86^ like *C. elegans* are Nile Red^87^ and BODIPY.^88^ Nile Red and BODIPY operate under the 450–500 nm excitation and ∼520 nm emission. Notably, despite their widespread use, concerns have been raised for their reliability. Specifically, BODIPY undergoes spectral shifts from blue excitation–green emission to green excitation–red emission due to dye dimerization states under different biological conditions.^89^ Additionally, both Nile Red and BODIPY were found to accumulate in lysosome-related organelles (LROs) including peroxisomes, leading to inconsistencies between dye signals and lipid levels, observed in both mammalian cells^90^ and live animals such as *C. elegans*.^91^ To address these issues, advanced fluorescent small-molecule probes have been developed based on coumarin, 1,8-naphthalimide, 3-hydroxyflavone, benzoxadiazole, etc.^92^ In fixed animals, Oil Red O (ORO) staining is considered more reliable and precise for quantitative lipid assessment. For instance, this method has been applied in *C. elegans*, adipocytes, other cell types,^93^ and tissue sections.^94^ ORO-stained images can be visualized and interpretable with the naked eye, although the use of microscope with appropriate magnification is recommended for high-resolution view and accurate quantification.

#### 5.1.3 Combination of Small-Molecule Dyes and Fluorescence Protein Tagging

Small-molecule dyes and genetically encoded biosensors are commonly used together to validate the localization of proteins of interest. For example, ALDH3B1 tagged with eGFP was analyzed through colocalization with Oil Red O staining for LDs and immunofluorescence detection of ABHD5-FLAG. Similarly, ARL8B, a protein involved in mediating LD contact and delivery to lysosomes, is located in LD and lysosomes based on a combined analysis of mCherry-ARL8B expression, BODIPY staining for LDs, and LysoTracker staining for lysosomes.^95^ Furthermore, advanced imaging techniques such as fluorescence recovery after photobleaching (FRAP) and fluorescence-lifetime imaging microscopy (FLIM) are regularly integrated into these combination imaging regimens. For instance, FRAP analysis in live HuH-7 cells revealed that GFP-PLIN2 does not rapidly diffuse between LDs,^96^ and caveolin-1-GFP mobility is lower than perilipin in 3T3-L1 adipocytes.^97^ FLIM in combination with BODIPY-C_12_, LD viscosities were visualized.^98^ FLIM leveraging a π-extended fluorescent coumarin analog demonstrates visualization of LDs in cells and hepatocytes of live mice.^99^ The use of FP-based and small-molecule-dyes-based FRET (Förster resonance energy transfer) pairs, respectively, detect ABDH5 ligands in LDs^100^ and interactions between LDs and lysosomes.^101^

#### 5.1.4 Electron Microscopy

Electron microscopy (EM) is widely used for examining the single-membrane structure^102^ of LDs, the morphology,^103^ and their spatial interactions with other organelles.^104^ For example, EM offers visual analyses of direct interactions of LDs with autophagosomal membranes^105^ and engulfment of LD components to lysosomes in mouse hepatocytes.^26^ Cryo-EM detection reveals a liquid-crystalline phase in LDs related to cellular states and organelle association, hinted the pathological changes of LDs under specific conditions.^106^ Although electron microscopy provides intricate detail, it is limited to fixed specimens, making it less applicable for studies requiring dynamic or live-cell interrogations.^107^ The approach is time and labor intensive, and less widely accessible compared to, for instance, confocal microscopy, requiring special technical competence, often with a hefty price.

#### 5.1.5 Other Biophysical Methods

Imaging-based tools remain a go-to approach for visualizing LDs and underlying proteome-level changes. Nonetheless, understanding compositional lipid species within LDs remains largely out of reach, since dye-tagging of lipid molecules is significantly invasive, altering their physicochemical properties, functions, and trafficking. Small-molecule-based mass spectrometry (MS) and nuclear magnetic resonance spectroscopy (NMR) approaches are classically used to characterize lipid species within isolated LD extracts. More recently, several methods to map the lipid composition with spatial resolution have emerged. Polarized light microscopy, for instance, leverages the anisotropic birefringent properties of cholesteric liquid crystals gained upon illumination with polarized light, enabling the detection of heterogeneity in LD lipid composition.^108^ Another powerful technique is stimulated Raman scattering (SRS) microscopy, which focuses on the unsaturated C = C bonds in lipids, allowing the label-free imaging of lipid-rich structures.^109^ SRS thus proves particularly useful in distinguishing saturated and unsaturated fatty acids, with applications demonstrated in cells, yeast,^110^ tissues, and live animals like *C. elegans*.^111^ These studies show that monounsaturated fatty acids are upregulated LDs and peroxisomes in *C. elegans*, contributing to lifespan extension.^80^ Additionally, matrix-assisted laser desorption/ionization (MALDI) based MS imaging, provides detailed information on lipid species albeit in nonlive samples such as tissue sections of the brain.^112^

### 5.2 Proteomics-Based Strategies to ID LD-Associated Proteins

#### 5.2.1 LD Protein Profiling

Proteomics is a powerful approach for identifying unknown proteins, and under careful and rigorous experimental design, for understanding protein functions. LD components, including the LD-associated proteins can be isolated through cell lysis and ultracentrifugation. Indeed, many LD proteins have been identified through LD proteomics, including DHS-3 in *C. elegans*, endosome sorting complexes required for transport (ESCRT) in yeast,^113^ and small GTPase Rabs.^114,115^ Proteomics studies can also investigate protein recruitment under specific conditions^116^ to study the functions^115,117^ of specific LD proteins. For example, proteins involved in fatty acid catabolism or xenobiotic metabolism including cytochrome P450 and apolipoproteins are enriched in the LD fraction of hepatocytes following high-fat feeding to mice.^82^ However, most available LD-protein-based proteomics data sets have not leveraged quantitative mass-spectrometry workflows: the abundance could thus be biased by sample extraction and processing steps. In addition, lysis and LD protein extraction destroy LD microenvironment, and the procedure is further prone to protein leakage from LDs during isolation steps. The approach has limited ability to track dynamic protein turnover on LDs in live cells. Target identification results derived from classical LD-proteomics approaches thus necessitate additional rigorous mechanistic validations. Genetically encoded FP tagging of identified proteins and imaging confirmation of their LD-association against known LD-markers is the most common validation approach, but FP-tagging could also introduce potential artifacts as discussed above.

#### 5.2.2 Proximity Labeling and Interactome Mapping

Recent advancements in local-specific (proximity-based) proteomics technologies, e.g., BioID, TurboID, APEX2,^118^ have allowed for a more targeted study of the LD proteome. Based on the construct of APEX2-PLIN2,^119^ this method identifies multiple hydroxysteroid dehydrogenase (HSD) enzymes, redox-relevant enzymes, and ubiquitinylation-related proteins located in LDs.^119^ Proximity-based interactome mapping based on APEX2-PLIN1 constructs identifies interactions between PLIN1 and 4 members of the 14–3–3 proteins.^120^ However, because LDs are closely related to lipid peroxidation and oxidative stress, the H_2_O_2_ treatment used in APEX2 may alter LD behavior under stress, potentially affecting the readouts. Other proximity-based platforms without the external H_2_O_2_ treatment are thus likely more suitable for LD studies, such as Bio-ID or Turbo-ID. Nonetheless, such platforms provide limited information on the functions of LD proteins or the downstream regulation of specific protein/protein–small-molecule interactions. In addition to biotinylation-based interactome mapping, affinity enrichment combined with quantitative MS workflows offers another powerful approach for studying LD-associated proteins. For instance, using GFP-Trap enrichment of FP-PLIN5, followed by MS analysis, mitochondrial protein FATP4 was identified to interact with PLIN5 and promote LD-to-mitochondria fatty acid transport.^70^

#### 5.2.3 Activity-Based Protein Profiling (ABPP)

In the context of LDs involved in transferring, isolating, and clearing lipid peroxidation products,^54,121^ understanding the physiological nuances of how various endogenous reactive small molecules interact with LD proteins and regulate LD biology is critical. ABPP is a functional, reactivity-based, proteomic technology that profiles protein targets of a specific small-molecule ligand. The most commonly deployed comparative ABPP profiling quantitatively maps potential ligandable targets by indirectly scoring the remaining non-ligand-bound pools of protein-targets/sites, using broadly reactive proxy electrophiles, e.g., iodoacetamide. As discussed in the section: “LD functions” (*vide supra*), several lipid peroxidation products house electrophilic motifs, that could modify protein-cysteines to regulate cell activities. For instance, HNE, as the representative lipid peroxidation product from PUFA, can be incorporated to LD from the membrane, for eliciting stress response that protects the biomembrane system. An indirect ABPP profiling of protein targets of lipid-peroxidation products such as HNE shows HNEylation of the kinase ZAK, that inhibits JNK pathway downstream.^122^ However, limited spatiotemporal resolution of the ABPP platform against broad reactivity of electrophilic lipid-peroxidation products, renders it challenging to study precise ramifications of reactive molecules such as HNE, especially for compartmentalized proteins and those undergoing dynamic subcellular trafficking, such as LD or LD-related proteins.

### 5.3 Localis-REX and T-REX: Simultaneous Function-Guided Proximity Mapping and Precision Signaling Interrogations into Direct Targets of RES and RES-Regulated LD Functions

In 2018, a function-guided live-cell-based proteomics platform, G-REX, was introduced to quantitatively identify proteins sensitive to bioactive lipid-derived electrophiles such as HNE.^123^ G-REX gained local specificity in 2022^124^ (termed Localis-REX), which allows for controlled localized generation of specific electrophiles and quantitative mapping of potential electrophile-responsive native protein targets in specific subcellular compartments. More recently, an inaugural development of applying Localis-REX in whole live animals, *C. elegans*, in an organ-specific (OS) manner, was achieved. Applications of OS-Localis-REX have improved locale-specific understanding of how gut-specific HNE upregulation alters global LD abundance and animal stress management.^125^

**Figure 5:**
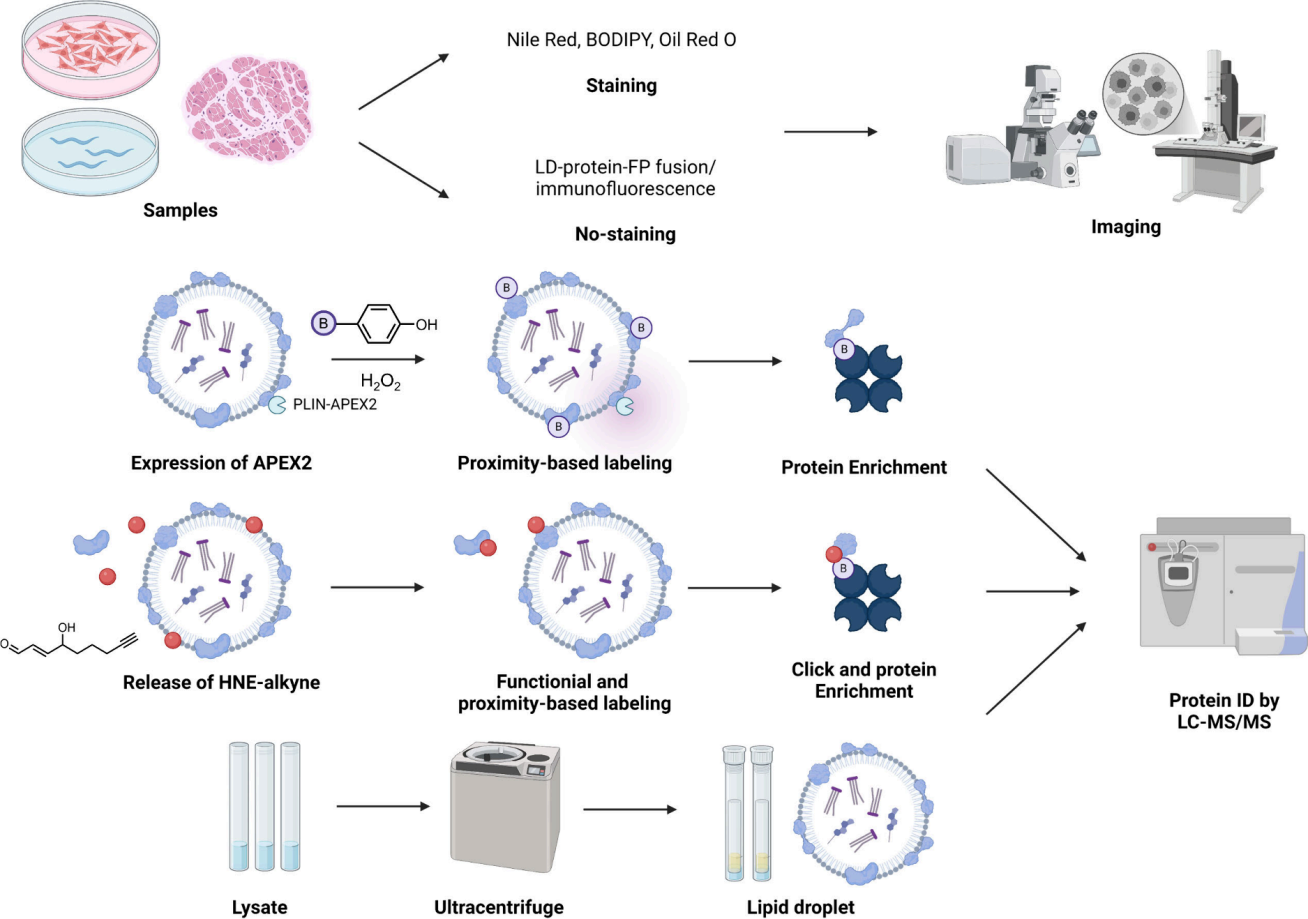
Platforms/technologies to study the LDs. The lipid droplets in cells, tissues, and transparent model organisms can be imaged. Typical approaches include the use of Nile Red, BODIPY, Oil Red O staining, and fluorescent protein fusion or immunofluorescence. Mass-spectrometry-based protein identification also constitutes a powerful platform for identifying unknown LD proteins. Proximity labeling tools (e.g., APEX2, top) have been used for LD-specific protein identification. RES-function-guided proximity mapping (Localis-REX generating localized HNE, middle, also see text) could be applied for the first-time identification of spatiotemporal HNE-responsive LD-specific proteins. Traditional methods rely upon isolating various LD components by ultracentrifugation (bottom).

It is in the authors’ opinions and outlooks that by applying Localis-REX targeted to LDs/LD-proteins, it would be possible to identify key LD-associated electrophile-responsive proteins, and mechanistically investigate, for instance, poorly understood precision signaling roles of HNE, and other relevant electrophiles, in LD-associated lipid metabolism and underlying pathways, including ferroptosis. Furthermore, by feeding forward newly identified Localis-REX-enabled HNE-function-guided hits to the sister technology, T-REX (Figure 5), the resulting Localis-REX–T-REX tandem technology could help clarify how LDs protect cells from lipid peroxides, crucial triggers of important processes, such as ferroptosis and oxidative stress-related disease contexts. Moreover, by targeting Localis-REX-mapping to additional organelles, specifically, mitochondria or the ER (where LDs originate), how ROS generated in mitochondria or ER stress affects the electrophile-guided functions of LD-proteins and their turnover mechanisms could be deconstructed. Last but not least, Localis-REX is potentially useful to resolve poorly understood lipid-associated mechanisms underpinning multiple disease etiologies, such as NAFLD or NDs, in which lipid dysregulation and elevated lipid peroxidation products are implicated. These tools can help fill crucial missing knowledge voids surrounding the role of RES signaling in LD dysregulation. New mechanistic knowledge could support the development of novel targeted therapies based on native electrophile-motif-harboring covalent small-molecule modulators.

## 6 Outlook

LDs have gained more and more attention in recent years. Novel LD proteins, LD regulatory factors/pathways, the importance of LDs in numerous diseases and stress response and surveillance mechanisms, and their broader roles in cellular metabolism, have expanded our understanding of these unique organelles. However, many questions remain unanswered. The dynamic regulatory mechanisms controlling LD turnover in different cell types and specific pathophysiological contexts need further investigations. Notably, as we discussed above, contextual roles of many key LD-regulators, e.g., AMPK, mTOR, lipin, and SERBP, remain controversial. These data underscore the level of complexity involved as well as precision tools required in accurate and comprehensive understanding of LD regulation. Precise mechanistic links between measurable phenotypic changes (e.g., cellular LD levels, animal behaviors, cell growth rate, etc.) and changes in specific proteins (expression, PTMs, interactomes, and so on), and mechanisms of conserved importance across taxa, altogether remain limited for the most part. In humans, LDs have emerged as critical players in metabolic diseases, cancer, and neurodegenerative disorders. Targeting LD dynamics and their associated pathways for therapeutic purposes is promising. For example, manipulating LD regulators and the enzymes involved in lipid metabolism may provide novel interventions. Finally, having discussed the latest findings and emerging approaches in studying LDs, it is in the authors’ opinion that precision medicine developments, enabled through identification of druggable LD-associated proteins, would benefit from new and improved precision technologies to study LD chemical biology. We hope the perspective draws attention and interests of researchers both in similar disciplines and further afield, and stimulates increased motivation toward the research into LDs and broader lipid-guided proteome signaling and organismal regulation.
